# The role of the triglyceride (triacylglycerol) glucose index in the development of cardiovascular events: a retrospective cohort analysis

**DOI:** 10.1038/s41598-019-43776-5

**Published:** 2019-05-13

**Authors:** Sangsang Li, Bingxin Guo, Huanan Chen, Zhan Shi, Yapeng Li, Qingfeng Tian, Songhe Shi

**Affiliations:** 1 Department of Epidemiology and Biostatistics, College of Public Health, Zhengzhou University, Zhengzhou, Henan P.R. China; 2 Department of Pharmacy, Zhengzhou People’s Hospital, Zhengzhou, Henan P.R. China; 3 Department of Neurology, The First Affiliated Hospital of Zhengzhou University, Henan, P.R. China; 4 Department of Social Medicine, College of Public Health, Zhengzhou University, Zhengzhou, Henan P.R. China

**Keywords:** Cardiovascular diseases, Epidemiology, Risk factors

## Abstract

This study aimed to evaluate the role of the triglyceride (triacylglycerol) glucose (TyG) index in predicting and mediating the development of cardiovascular disease (CVD). This cohort study included 6078 participants aged over 60 years who participated in a routine health check-up programme from 2011 to 2017. The competing risk model, cox regression model and multimediator analyses were performed. TyG was calculated as ln [fasting triglyceride (mg/dl) × fasting plasma glucose (mg/dl)/2]. During a median 6 years of follow-up, 705 (21.01/1000 person-years) CVD events occurred. In fully adjusted analyses, quartiles 3 and 4 versus quartile 1 of TyG index (adjusted subhazard ratios [SHRs] 1.33 [95% CI: 1.05–1.68] and 1.72 [1.37–2.16]) were associated with an increased risk of CVD events. The continuous time-dependent TyG remained significant in predicting CVD events (adjusted hazard ratios [HR] 1.43 [1.24–1.63]). The adverse estimated effects of body mass index (BMI) or resting heart rate (RHR) on CVD mediated through the joint effect of the baseline and follow-up TyG index. In addition, an effect mediated only through the follow-up TyG existed (*P* < 0.05). Thus, it is necessary to routinely measure the TyG. The TyG index might be useful for predicting CVD events in clinical practice.

## Introduction

Cardiovascular disease (CVD) was responsible for approximately 11.1 million global deaths among the elderly in 2016, and it is the leading cause of mortality in China^1^. Hence, it is essential to prevent the development of CVD to prevent premature deaths. Before the manifestation of CVD, the detection of insulin resistance (IR) in apparently healthy subjects has clinical implications in the prevention of CVD development^2^. However, the hyperinsulinaemic-euglycaemic clamp, which is the gold-standard test for IR, is time-consuming, costly and complex^3^. Another alternative approach is to use the triglyceride glucose (TyG) index, which is calculation using the levels of triglycerides (TG or triacylglycerols [TAG]) and fasting plasma glucose (FPG). The TyG index, an inexpensive and reliable surrogate indicator, has been proven to be strongly correlated with the identification of IR^3–6^. Furthermore, the TyG index has been shown to predict CVD events in an apparently healthy Caucasian population^2,7^. Nonetheless, the findings regarding the relationship between the TyG index and CVD events remain limited, with a relatively small pool of studies. To the best of our knowledge, the association between the TyG index and CVD events among the elderly has not been studied in China.

Additionally, body mass index (BMI) and resting heart rate (RHR) have gained interest as potential clinical measurements and prognostic indicators for CVD^8–13^. However, the role of the TyG index in the link between BMI/RHR and CVD events is unclear. Mediation analysis could clarify the role of the TyG index, if any, in the relationship between BMI/RHR and CVD events.

Therefore, the aims of the present study were to specifically explore the link between the TyG index and incident CVD and to determine whether the TyG index is a mediator of the possible mechanisms underlying the association between BMI/RHR and CVD events. The hypothesis is that a higher TyG index is associated with an elevated risk of CVD among elderly people, and the relationship between BMI/RHR and CVD events is partly or fully mediated by the TyG index.

## Results

### Baseline characteristics of study participants

The baseline characteristics of the 6078 participants included in the study stratified by quartiles of the TyG index are summarized in Table 1. The mean (standard deviation, SD) age of the participants was 70.45 (6.79) years. Individuals were observed for a mean of 5.52 years (median of 6 years). During a mean of 33557.52 person-years of follow-up, 705 CVD events occurred (overall incidence: 21.01/1000 person-years), of which 500 (14.90/1000 person-years) were coronary heart disease (CHD) events and 234 (6.97/1000 person-years) were cerebrovascular disease events. In these participants without baseline CVD history, 26 individuals died due to CHD, 48 individuals died due to cardiovascular disease, and 183 individuals died due to non-CVD causes. The TyG index quartiles were higher in men than in women and in participants with higher BMIs, higher RHRs, higher systolic blood pressure (SBP) and less physical exercise than in individuals with lower BMIs, lower RHRs, lower SBP, and more physical exercise, respectively. Furthermore, a higher prevalence of type 2 diabetes mellitus (T2DM) was found in participants with higher TyG index quartiles.

**Table 1:** Baseline characteristics of the included participants according to quartiles of baseline TyG index.

Variables	TyG index				*P* value for trend
	Quartile 1 (<8.32) n = 1523	Quartile 2 (8.32–8.61) n = 1521	Quartile 3 (8.61–8.89) n = 1515	Quartile 4 (≥8.90) n = 1519	
Age (years), mean (SD)	70.32 ± 6.83	70.81 ± 6.95	70.36 ± 6.76	70.31 ± 6.61	0.540
Women, n (%)	820 (53.84)	734 (48.26)	706 (46.60)	592 (38.97)	<0.001
Living alone, n (%)	369 (24.23)	430 (28.27)	418 (27.59)	426 (28.04)	0.034
Current smoking, n (%)	193 (12.67)	201 (13.21)	212 (13.99)	239 (15.73)	0.012
Alcohol consumption, n (%)	128 (8.40)	193 (12.69)	209 (13.80)	224 (14.75)	<0.001
Exercise, n (%)	181 (11.88)	152 (9.99)	142 (9.37)	127 (8.36)	<0.001
BMI (kg/m^2^), mean (SD)	22.98 ± 2.77	23.55 ± 3.01	23.69 ± 3.00	24.75 ± 3.42	<0.001
RHR (beats per minute), mean (SD)	73.80 ± 6.44	74.42 ± 7.51	74.54 ± 7.89	75.41 ± 8.39	<0.001
SBP (mmHg), mean (SD)	130.77 ± 16.58	133.10 ± 17.69	133.97 ± 18.44	138.53 ± 20.34	<0.001
TG (mmol/L), median (IQR)	0.85 (0.67–1.00)	1.23 (1.12–1.34)	1.50 (1.40–1.60)	2.23 (1.80–2.87)	<0.001
FPG (mmol/L), median (IQR)	4.60 (4.18–5.05)	4.96 (4.60–5.28)	5.30 (4.94–5.65)	5.60 (5.08–6.00)	<0.001
HDL-C (mmol/L), median (IQR)	1.38 (1.20–1.79)	1.36 (1.20–1.74)	1.30 (1.20–1.50)	1.30 (1.12–1.58)	<0.001
LDL-C (mmol/L), median (IQR)	2.61 (1.96–3.20)	2.50 (2.10–3.11)	2.60 (2.25–3.20)	2.80 (2.27–3.24)	<0.001
TyG index, median (IQR)	8.07 (7.82–8.21)	8.49 (8.40–8.56)	8.75 (8.68–8.81)	9.16 (9.00–9.43)	<0.001
T2DM (%), n (%)	132 (8.67)	139 (9.14)	181 (11.95)	265 (17.45)	<0.001

*P* value for trend of linearity was obtained from linear regression for continuous variables and logistic regression for categorical variables. SD = standard deviation; IQR = interquartile range; TyG = triglyceride glucose; BMI = body mass index; RHR = resting heart rate; SBP = systolic blood pressure; TG = triglyceride; FPG = fasting plasma glucose; HDL-C = high-density lipoprotein cholesterol; LDL-C = low-density lipoprotein cholesterol; T2DM = type 2 diabetes mellitus.

### Association between the TyG index and the development of CVD

Taking quartile 1 of the TyG index as the reference group, the adjusted subhazard ratios (SHRs) and hazard ratios (HRs) for CVD were significantly higher in quartile 4 of the TyG index (Table 2 and Suppl. Table S1). Specifically, in competing-risks model 2 for CVD events, the corresponding SHRs for quartile 2, 3 and 4 were 0.99 (95% confidence interval [CI]: 0.77–1.28), 1.33 (1.05–1.68), and 1.72 (1.37–2.16), respectively. Risk for CHD and CVD was significantly higher across increasing quartiles of TyG (*P* for trend of linearity <0.001). However, further adjustment (model 2 and model 3) did not have a significant impact on the association of TyG with cerebrovascular diseases.

**Table 2:** Association of cardiovascular disease, coronary heart disease, and cerebrovascular disease events competing with non-event death across quartiles of triglyceride glucose.

Incident Event	Model	TyG index				
		Quartile 1 (<8.32) n = 1523	Quartile 2 (8.32–8.61) n = 1521	Quartile 3 (8.61–8.89) n = 1515	Quartile 4 (≥8.90) n = 1519	*P* for trend of linearity
CVD	CVD	121 (7.94)	128 (8.42)	177 (11.68)	279 (18.37)	<0.001
	Non-CVD death	41 (2.69)	47 (3.09)	54 (3.56)	41 (2.70)	NA
	Model 1	Reference	1.05 (0.82–1.35)	1.49 (1.18–1.88)	2.38 (1.92–2.96)	<0.001
	Model 2	Reference	0.99 (0.77–1.28)	1.33 (1.05–1.68)	1.72 (1.37–2.16)	<0.001
	Model 3	Reference	1.00 (0.80–1.25)	1.17 (0.94–1.45)	1.61 (1.31–1.99)	<0.001
CHD	CHD	71 (4.66)	86 (5.65)	118 (7.79)	225 (14.81)	<0.001
	Non-CHD death	56 (3.68)	54 (3.55)	69 (4.55)	52 (3.42)	NA
	Model 1	Reference	1.19 (0.87–1.64)	1.68 (1.25–2.26)	3.28 (2.51–4.30)	<0.001
	Model 2	Reference	1.14 (0.83–1.57)	1.50 (1.11–2.03)	2.32 (1.75–3.08)	<0.001
	Model 3	Reference	1.22 (0.93–1.60)	1.26 (0.96–1.66)	2.05 (1.58–2.64)	<0.001
Cerebrovascular disease	Cerebrovascular disease	52 (3.41)	45 (2.96)	63 (4.16)	74 (4.87)	0.006
	Non-cerebrovascular disease death	46 (3.02)	50 (3.29)	63 (4.16)	50 (3.29)	NA
	Model 1	Reference	0.87 (0.58–1.29)	1.24 (0.86–1.80)	1.46 (1.02–2.09)	0.008
	Model 2	Reference	0.81 (0.54–1.21)	1.12 (0.77–1.63)	1.12 (0.78–1.62)	0.308
	Model 3	Reference	0.67 (0.45–0.99)	1.12 (0.79–1.59)	1.20 (0.84–1.72)	0.183

Values are n (%) or subhazard ratio (95% confidence interval) using the first (lowest) quartile as the reference. Model 1 was adjusted by age and sex; model 2 was model 1 plus living alone, current smoking, alcohol consumption, exercise, body mass index, resting heart rate, systolic blood pressure, high-density lipoprotein cholesterol, low-density lipoprotein cholesterol, and diabetic status; model 3 was model 2 with time-varying repeated measures of TyG. *P* values for linear trend across TyG quartiles were evaluated by a median value within each quartile as a continuous variable. TyG = triglyceride glucose; CVD = cardiovascular disease; CHD = coronary heart disease; NA = not applicable.

In the continuous analysis (Suppl. Table S2), even after adjustment for time-varying TyG values, the TyG was significantly associated with the risk of CVD (HR: 1.43; 95% CI: 1.24–1.63) and CHD events (HR: 1.63; 95% CI: 1.39–1.90).

### Results of sensitivity analysis

**Figure 1:**
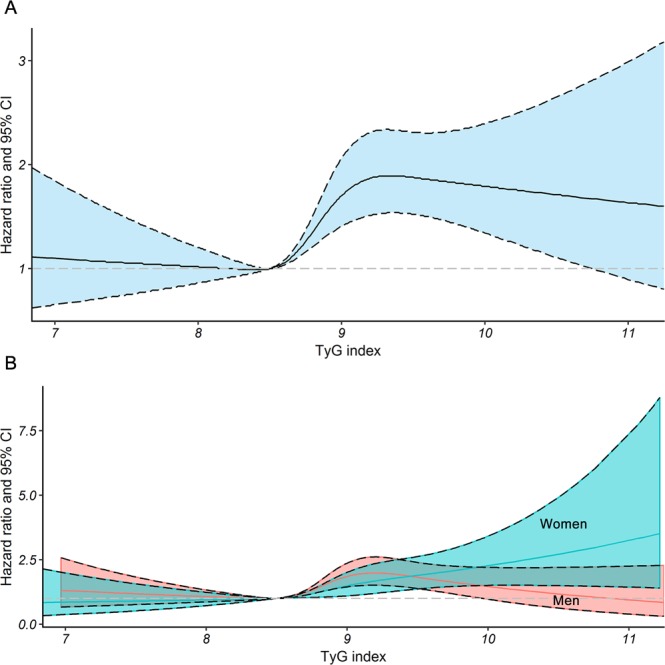
Nonlinear relationship between incremental increases in the TyG index and the risk of CVD. Multivariate adjusted hazard ratios of CVD associated with the baseline TyG index values for (**A**) the overall population (**B**) and the subgroups stratified by sex in restricted cubic splines, adjusted for age, sex, living alone, current smoking, alcohol consumption, exercise, body mass index, resting heart rate, systolic blood pressure, high-density lipoprotein cholesterol, low-density lipoprotein cholesterol, and type 2 diabetes mellitus. TyG = triglyceride glucose; CVD = cardiovascular disease; CI = confidence interval.

There was a nonlinear association between the continuous value of the TyG index and the risk for CVD in the fully adjusted cubic spline model. An increasingly higher TyG index value ranging from 8.50 to 10.75 was associated with a higher risk of CVD (Fig. 1A). In splines for the sex subgroups, the TyG levels were more likely to trend towards a higher risk of CVD among women rather than among men when a TyG value was larger than 9.53 (Fig. 1B). However, the sex differences in the relationship between TyG and CVD risk in the extremely high level of the TyG index needed to be interpreted with caution due to the small sample size.

The main results in the subgroup analyses were consistent with the findings from the total population (Suppl. Table S3). The multiple competing-event results showed that TyG was associated with non-fatal CHD events (Suppl. Table S4). Participants lost to follow-up (n = 412) vs participants included in the study (n = 6078) were younger and had a lower SBP and a higher high-density lipoprotein cholesterol (HDL-C) (Suppl. Table S5). After a multiple imputation of missing data on incident CVD in the loss, the results showed that the HR in model 2 remained significantly associated, with a value of 1.30 (95% CI: 1.03–1.64) for those in the third quartile, and 1.68 (95% CI: 1.34–2.10) for those in the highest quartile.

### ROC analyses for TyG and BMI to predict the incident risk of CVD

The area under the receiver operating characteristic curves (AUCs) for the TyG index and BMI were 0.612 (95% CI: 0.590–0.635) and 0.611 (95% CI: 0.589–0.632), respectively (Suppl. Fig. S1). No significant differences were observed between the AUCs for the TyG index and BMI (*P* = 0.898). The best TyG value for diagnosis of CVD was 8.50 (sensitivity: 0.745, specificity: 0.404).

### The role of the TyG index in mediating the effect between the RHR or BMI and CVD events

**Figure 2:**
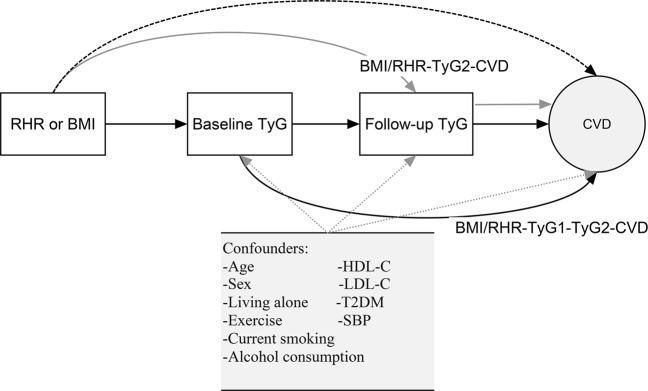
Decomposition of the total effect of RHR or BMI on the risk of incident CVD into the path-specific effects mediated by the baseline and follow-up TyG index values. The three path-specific effects are as follows: BMI/RHR-CVD, the effect of baseline BMI/RHR on the risk of CVD events independent of the baseline and follow-up TyG index values is shown with the dashed arrow in black; BMI/RHR-TyG1-TyG2-CVD, the effect of baseline RHR/BMI on the risk of CVD events mediated through baseline TyG index values and possibly through follow-up TyG index values is shown with the solid arrows in black; and BMI/RHR-TyG2-CVD, the effect of baseline BMI/RHR on the risk of CVD events mediated only through follow-up TyG index values is shown with the solid arrows in grey. The dotted arrows in grey indicate potential effects of multiple confounders on the mediators and the outcome. BMI = body mass index; RHR = resting heart rate; CVD = cardiovascular disease; TyG1 = baseline triglyceride glucose; TyG2 = follow-up triglyceride glucose; SBP = systolic blood pressure; HDL-C = high-density lipoprotein cholesterol; LDL-C = low-density lipoprotein cholesterol; T2DM = type 2 diabetes mellitus.

Interestingly, statistically significant associations were also found between the per 1-unit increase in RHR or BMI and the occurrence of CVD in the fully adjusted model 2. Mediation analysis in terms of estimates of how much of the observed relationship between BMI/RHR and CVD events could be attributable to indirect effects of the baseline TyG index value and the follow-up TyG index value was explored (Table 3 and Fig. 2). In the mediation analysis using the Cox model, a per 10-beat increase of RHR increased the risk of incident CVD through the mechanism of RHR-TyG1 (baseline TyG)-TyG2 (Follow-up TyG)-CVD with a hazard ratio of 1.014 (95% CI: 1.007–1.024). Moreover, there existed an effect mediated only through the follow-up TyG from the test in RHR-TyG2-CVD path (*P* = 0.018). Similarly, both the baseline and follow-up TyG accounted for the excess effect of per 1-unit increase of BMI (indirect effect for both: HR 1.010; 95% CI: 1.006–1.015) on CVD. The results from the Aalen additive hazard model (Table 3), which was regarded as a sensitivity analysis, were in line with those from the Cox proportional hazard model.

**Table 3:** Excess effects and total effects of per 10-beat increase of RHR or per 1-unit increase of BMI on CVD incidence mediated through the baseline and follow-up TyG index.

Path-specific mediation effects	Aalen Model		Cox model	
	Hazard difference (per 1000 person-year) (95% CI)	*P*	Hazard ratio (95% CI)	*P*
**For RHR and CVD**				
RHR-CVD	4.225 (2.372, 6.078)	<0.001	1.238 (1.130, 1.357)	<0.001
RHR-TyG2-CVD	0.241 (0.071, 0.440)	0.021	1.012 (1.003, 1.021)	0.018
RHR-TyG1-TyG2-CVD	0.278 (0.120, 0.475)	0.009	1.014 (1.007, 1.024)	0.004
Total effect	4.744 (2.871, 6.616)	<0.001	1.271 (1.159, 1.394)	<0.001
**For BMI and CVD**				
BMI-CVD	1.592 (1.079, 2.104)	<0.001	1.081 (1.056, 1.106)	<0.001
BMI-TyG2-CVD	0.193 (0.124, 0.272)	<0.001	1.009 (1.006, 1.013)	<0.001
BMI-TyG1-TyG2-CVD	0.196 (0.100, 0.298)	<0.001	1.010 (1.006, 1.015)	<0.001
Total effect	1.981 (1.466, 2.496)	<0.001	1.102 (1.078, 1.127)	<0.001

BMI = body mass index; RHR = resting heart rate; TyG1 = baseline triglyceride glucose; TyG2 = follow-up triglyceride glucose; CVD = cardiovascular disease.

## Discussion

In this large-scale cohort of older participants who underwent regular health check-ups, we found that increasing TyG index values were significantly associated with an increased risk of CVD. However, TyG was not independently associated with the risk of cerebrovascular disease. Our analyses further revealed that significant excess risks for CVD due to BMI/RHR were mediated through the TyG index. To the best of our knowledge, this is the first study to explore the role of the TyG index as a potential predictor and mediator for CVD events among the elderly.

The finding that the TyG index is associated with increased risk of CVD is supported by previous studies^2,7^. As noted earlier, the TyG index is a promising surrogate index for IR and a candidate marker for discriminating metabolic diseases^4,5,14^. Moreover, IR is regarded as the central event in incident T2DM^15^. Therefore, a wealth of previous studies demonstrated that the TyG index is a valuable predictor for T2DM^3,6,16^. IR is also considered strongly correlated with the risk of developing CVD^17–19^. This indicates that it may be plausible to use the TyG index as a surrogate indicator for IR to identify its role in CVD development. In this respect, our study shed light on the significant relationship between the TyG index and the risk of CVD in the elderly and the nonlinear dose-dependent association of increased TyG index values with a higher risk of incident CVD, which was in line with the study by Sanchez-Inigo *et al*.^2^.

There is a biologically plausible mechanism underlying the significant association between the TyG index and incident CVD. There is a strong relationship between insulin resistance and endothelial dysfunction^20,21^. Endothelial dysfunction, which is ascribed to IR, contributes to CVD, including atherosclerosis and coronary artery disease^22^. In addition, during injury, the metabolic stability of energetic substrates utilized by the heart can be impaired by IR, producing lipotoxicity in the heart^23^. Therefore, as a surrogate index for IR, the TyG index is a possible factor that is associated with incident CVD.

The current study found that TyG is a stronger risk factor for CVD events in women than in men when a TyG value is larger than 9.53. Similarly, several studies found that there exists “female disadvantage” in transition from normoglycemia to impaired glucose tolerance and diabetes and the reason remains unknown^24–26^. There is not yet sufficient evidence on how sex difference can modulate the effects of TyG on cardiovascular risk. The sex differences in body composition and fat distribution might contribute to this difference^27^. In addition, although the lower age-specific CVD risk in women than in men can be attributable to the protective effect of sex steroid hormones in women, especially estrogen, estrogen supplementation does not reduce the risk of CVD in postmenopausal women^28^. Thus, it is plausible that there is no protective effect of estrogen in elderly women. Many other factors, such as genetic factors and sex chromosome genes asset can play a relevant role^29^.

BMI is a well-established risk factor for the development of T2DM and CVD. In the present study, the ability of TyG index to predict incident CVD was similar to this obesity-related marker for the elderly. It is noteworthy that the waist-to-hip (WHR) exhibited enhanced predictive capability compared to BMI^30,31^. The WHR is a simple measure of central obesity and calculated as waist measurement divided by hip measurement. However, our research did not include the waist-to-hip ratio because of the lack of baseline measurement of hip circumference among the participants. Further study on the comparison of the predictive role of WHR and TyG needs to be explored.

Additionally, our results indicated that both the baseline and follow-up TyG index values mediated the relationship between BMI and CVD events. The reduction in the absolute value of the HR for the effect of BMI on CVD demonstrated that TyG was a partial mediator of the relationship between BMI and CVD. Similarly, Low *et al*. found a partial mediating role of the TyG index in the association between BMI and the development of T2DM^16^. Overweight and obesity (according to BMI categories) have harmful effects on CVD risk, and the underlying mechanism has been thoroughly investigated. Adipose tissue increases basal lipolysis and releases free fatty acids (FFA), interleukins and cytokines that drive cardiac dysfunction by accelerating atherosclerotic processes and modifying factors associated with inflammation and endothelial and coagulation dysfunction^32,33^. The increase in FFA due to overweight or obesity can trigger IR, which further inhibits insulin signalling and insulin-stimulated glucose uptake in skeletal muscles and increases glucose delivery by the liver^34,35^. Furthermore, inflammatory factors associated with obesity promote the processes of lipolysis and hepatic triglyceride synthesis, as well as hyperlipidaemia induced by increased fatty acid esterifcation^19^. Hence, the TyG index, which includes TG and FPG in its formula, is a plausible link between BMI and the risk of CVD. For public health programmes, the mediating role of the TyG index in the association between BMI and CVD indicates that it is still important to emphasize body weight management and to encourage older people to focus on having their TG and FPG checked regularly.

The interesting finding of the association of RHR with CVD among elderly participants in our study was in line with the results of a published meta-analysis including 87 research studies that revealed that every 10-beat increase in the RHR was associated with a 15% higher risk of CVD^36^. At the same time, the fact that the RHR is an independent predictor of CHD and stroke has been quantitatively assessed^13^. Sympathetic overactivity, which is associated with IR and the progression of other processes, such as cardiac remodelling, atherosclerosis, and pro-arrhythmic effects, may explain the impact of RHR on the incidence of CVD^37^. Then, the hypothesis that IR due to sympathetic overactivity may be involved in a causal pathway between the RHR and the risk of CVD is reasonable. Nonetheless, this hypothesis has not been explored in a human study. Because the link between the TyG index and CVD was supported by the evidence that an increase in the RHR was related to CVD risk in our study, the TyG index might be regarded as being involved in the underlying mechanism. To illustrate this underlying mechanism, we used a mediation analysis and found that the association between the RHR and CVD was partially mediated by the TyG index. In this sense, lowering the RHR and tracking the concentrations of TG and FPG can contribute to preventing the development of CVD. Because the RHR-TyG1-TyG2-CVD path consists of two mediators, namely, the baseline and follow-up TyG, this pathway cannot rule out the possibility that the follow-up TyG index value is a mediator. Notably, incremental increases in the BMI/RHR increased the risk of CVD only via the mediating effect of the follow-up TyG index value (BMI/RHR-TyG2-CVD) were significant in this study. Therefore, we confirmed that the follow-up TyG is a mediator and excess effect caused by BMI/RHR-TyG1-TyG2-CVD path contains the effect mediated by both the baseline and follow-up TyG. The madiation results indicate the clinical implication that when abnormal RHR values exist in older individuals, intensive follow-up including the monitoring of TG and FPG concentrations should be considered.

The major strengths of the current study are the large number of elderly individuals and adjustment for main CVD risk factors. Moreover, we used mediation models with multiple mediators to characterize the possible role of the TyG index in the comprehensive mechanism underlying the risk of CVD. In addition, the main results remained robust after conducting several sensitivity analyses.

Several limitations should be acknowledged as well. Firstly, as in any observational study, our results cannot exclude influence due to unmeasured and residual confounding variables. However, we used the well-known risk factors for CVD as confounders. Secondly, we did not measure insulin levels to generate a homeostatic model assessment (HOMA), which is a validated and widely used marker for IR, and we did not assess glucose tolerance. Therefore, we cannot compare the predictive power for CVD between the TyG index and HOMA. Future studies are needed to compare the predictive role of the TyG index and HOMA for CVD risk. Thirdly, recall error in self-reported confounders was unavoidable. Fourthly, the majority of the elderly could not respond regarding their history of lipid-lowering therapy or antidiabetic drugs; thus, there was a lack of information on therapy in the data. Then, the current results might have a bias away from the null hypothesis. In the future, we will try to obtain access to medication records from hospitals to reduce this bias. Finally, distribution of some covariates in the participants lost to follow-up varying from the final cohort indicates that the missing follow-up data may affect statistical power or bias the findings. However, the initial sample size and retention rate were adequate to ensure sufficient power for the analyses. Participants lost to follow-up were younger. There is a typical Chinese culture of the increased prevalence of grandchild care provided by grandparents, thus the grandparents are likely to move to another location to accompany their grandchildren^38^. This may account for the difference in age and living alone between the loss and the final cohort.

In conclusion, an elevated TyG index value is significantly associated with an increased risk of developing CVD in elderly participants. In addition, both baseline and follow-up TyG index values mediate the association between BMI or RHR and the risk of CVD. Thus, the TyG index must be routinely measured. The TyG index is easily measurable and accessible for the evaluation of IR in clinical practice, and this index might be useful for identifying individuals who are at high risk of CVD events.

## Methods

### Study population

We performed a population-based, retrospective cohort analysis of participants of 60 years of age or older who entered the health check-up programme in Xinzheng and Xinmi City, Henan Province, in Central China, from January 2011 to December 2017. Health check-up costs were paid by the Chinese government. In total, there were 7690 participants eligible for the study. We excluded participants who had any of the following conditions: a history of CVD (n = 1040) or type 1 diabetes (n = 4) at the time of entry (n = 1044), extreme BMI values (>45 kg/m^2^) (n = 11), or having missing information on the TyG index values (n = 145). In the remaining 6490 participants, subjects who underwent only one screening examination without repeated measures and had missing information on the outcome during the follow-up period (n = 412) were also excluded. The final sample size was 6078. This study was approved by the ethics committee of Zhengzhou University in China. Given the retrospective nature of the research, the requirement for informed consent was waived.

### Data collection

The demographic information and clinical data of the included patients were collected at each visit. The demographic information of the population included age, sex (men/women), living alone (yes/no), medical history (hypertension, T2DM, CVD), current smoking (yes/no), daily alcohol consumption (yes/no) and strenuous exercise over 60 minutes at least once a week (yes/no). The definition of current smoking was that adults have smoked at least 100 cigarettes in their lifetime and they now smoke occasionally or every day. The clinical data included anthropometric measurements and laboratory investigations. The measurements including body height, weight, RHR and blood pressure were measured by trained personnel. The measurements for height and weight were performed with the subjects wearing light clothing without shoes. Blood pressure and the radial pulse rate were measured twice by an automatic sphygmomanometer (Omron HEM-7125, Kyoto, Japan) after subjects had rested in a seated position for at least 5 minutes, and the mean value of the two measurements was recorded. BMI was calculated as the weight in kilograms divided by the square of the height in metres. Blood samples were collected after a minimum fasting time of 8 hours and were analysed with an automatic biochemical analyser (DIRUI CS380, Changchun, China). Laboratory information, including FPG, HDL-C, low-density lipoprotein cholesterol (LDL-C) and TG, was used in this study. LDL-C was estimated using Friedewald’s formula^39^. The TyG index was calculated as the ln [fasting TG (mg/dl) × FPG (mg/dl)/2]^40^.

### Outcome definition

The outcome of interest in this study was CVD events, consisting of fatal and non-fatal CHD events and fatal and non-fatal cerebrovascular disease events^41^. These CVD events were identified from the data of the annual health check-up programme with a digital linkage to the hospital dataset for admissions. Death certificates were obtained as well. Outcomes were defined using codes from the International Classification of Diseases, Tenth Revision (ICD-10)^42^. CHD was identified using the codes I20-I25^43^, and cerebrovascular disease was identified using the codes I63-I66^2^.

### Statistical analysis

Continuous variables are presented as the mean (SD) or median (interquartile range [IQR]), and categorical variables are expressed as proportions. Study participants were divided into four groups based on the TyG quartiles. To obtain the *P* values for linear trend in baseline characteristics, the linear regression and logistic regression were used for continuous variables and categorical variables, respectively.

A Cox proportional hazard regression model was performed to estimate the HRs and 95% CIs for the quartiles of the TyG index in association with the occurrence of CVD, with quartile 1 of the TyG index as the reference group. Test for proportionality of hazards over time was performed and no major violations were observed (Suppl. Table S6). In primary CVD survival analyses, we assumed that individuals who died of causes other than CVD were still at risk of developing CVD. To address this biologically untenable assumption, we calculated cumulative incidences of CVD using competing-risks survival regression with the method of Fine and Gray^44^, and calculated the SHRs for CVD, accounting for the competing risk of nonevent death. The SHRs in the competing risk model could be interpreted similarly to HRs in Cox regression. There were three models (model 1, model 2 and model 3) for Cox regression and competing-risks regression. Model 1 controlled for covariates including age and sex, and model 2 adjusted for confounders including age, sex, living alone, current smoker, alcohol consumption, exercise, BMI, RHR, SBP, HDL-C, LDL-C, and T2DM. Model 3 was model 2 with time-varying repeated measures of TyG. For model 3, we reshaped the records at each visit, thus breaking the data set into time dependent parts. Then we could keep track of the time-varying effect of TyG. The linear term representing median value within each quartile of TyG index was used to obtain *P* values for trend of linearity.

For the sensitivity analyses, the potentially nonlinear relationship between the continuous TyG index and the occurrence of CVD was explored using restricted cubic spline models with four knots^45^. To test the robustness of this study, additional subgroup analyses on the basis of age, sex, and diabetes were also performed using the competing-risks regression. The multiple competing-event risks model was also performed. In addition, we took into account the effect of loss to follow-up. To compare the differences between the participants lost to follow-up and participants included in the study (Suppl. Table S5), the Welch two sample t-test and Mann-Whitney U test were used for continuous variables, and the chi-square test was used for categorical variables.

In addition, BMI is a well-known risk factor for CVD. Therefore, the AUCs were used to compare the the ability of baseline TyG index and BMI to predict the risk of CVD.

To assess whether the baseline and follow-up TyG index values mediated the BMI/RHR-CVD relationship via a possible mechanism involving IR and whether the TyG index is a potential mediator of the effect of BMI/RHR on CVD, we used the mediation analysis approach proposed by Huang *et al*. with an extension allowing for multiple mediators in the survival model^46^. This mediation model with two mediators showed a path-specific effect (Fig. 2). The mediation analysis used a Cox model to obtain the effect scale of HR and an Aalen additive hazard model to obtain the estimate of the difference in hazards^38^. In our study, the Aalen additive hazard model was considered a sensitivity analysis for the mediation analysis.

Mediation analysis was conducted using R version 3.5.0 (R Foundation for Statistical Computing, Vienna, Austria), and the other analyses were performed in STATA v12.0 (STATA Corp, College Station, TX, USA). The R code for performing mediation analyses in this study was shown in the supplementary file. Probability values for statistical tests, where two-tailed and *P*-values were less than 0.05, were regarded as significant.

## Supplementary information

Supplementary information
