# Supplementary material for: The role of the triglyceride (triacylglycerol) glucose index in the development of cardiovascular events: a retrospective cohort analysis
Source: Sci Rep. 2019 May 13;9:7320. doi: 10.1038/s41598-019-43776-5 (PMC6513983; doi:10.1038/s41598-019-43776-5)
Supplement: Supplementary file 1 — Supplementary information [file 41598_2019_43776_MOESM1_ESM.docx]

**SUPPLEMENTARY INFORMATION**

**The role of the triglyceride (triacylglycerol) glucose index in the development of cardiovascular events: a retrospective cohort analysis**

Sangsang Li, Bingxin Guo, Huanan Chen, Zhan Shi, Yapeng Li, Qingfeng Tian, and Songhe Shi

**SUPPLEMENTARY TABLES AND FIGURES**

**Supplementary Table S1.** Association of cardiovascular disease, coronary heart disease, and cerebrovascular disease events across quartiles of triglyceride glucose in Cox models.

| **Incident Event** |  | **TyG** | | | | ***P* for trend** |
| --- | --- | --- | --- | --- | --- | --- |
|  | **Model** | **Quartile 1 (<8.32)** | **Quartile 2 (8.32-8.61)** | **Quartile 3 (8.61-8.89)** | **Quartile 4 (≥8.90)** |  |
| CVD | Model 1 | Reference | 1.05 (0.82-1.35) | 1.50 (1.19-1.89) | 2.39 (1.93-2.96) | <0.001 |
|  | Model 2 | Reference | 1.00 (0.78-1.28) | 1.34 (1.06-1.70) | 1.73 (1.39-2.17) | <0.001 |
|  | Model 3 | Reference | 1.01 (0.81-1.26) | 1.18 (0.95-1.46) | 1.65 (1.34-2.02) | <0.001 |
| CHD | Model 1 | Reference | 1.21 (0.88-1.65) | 1.70 (1.27-2.28) | 3.28 (2.51-4.29) | <0.001 |
|  | Model 2 | Reference | 1.15 (0.84-1.58) | 1.53 (1.14-2.06) | 2.33 (1.77-3.08) | <0.001 |
|  | Model 3 | Reference | 1.23 (0.94-1.60) | 1.28 (0.97-1.67) | 2.08 (1.62-2.68) | <0.001 |
| Cerebrovascular disease | Model 1 | Reference | 0.87 (0.58-1.29) | 1.25 (0.86-1.80) | 1.47 (1.03-2.10) | 0.011 |
|  | Model 2 | Reference | 0.81 (0.54-1.21) | 1.13 (0.78-1.64) | 1.13 (0.78-1.65) | 0.291 |
|  | Model 3 | Reference | 0.67 (0.45-0.99) | 1.13 (0.80-1.60) | 1.23 (0.86-1.75) | 0.130 |

Values are hazard ratio (95% confidence interval) using the first (lowest) quartile as the reference. Model 1 was adjusted by age and sex; model 2 was model 1 plus living alone, current smoking, alcohol consumption, exercise, body mass index, resting heart rate, systolic blood pressure, high-density lipoprotein cholesterol, low-density lipoprotein cholesterol, and diabetic status; model 3 was model 2 with time-varying repeated measures of TyG. *P* values for linear trend across TyG quartiles were evaluated by a median value within each quartile as a continuous variable. TyG, triglyceride glucose; CVD, cardiovascular disease; CHD, coronary heart disease.

**Supplementary Table S2.** Association of cardiovascular disease, coronary heart disease, and cerebrovascular disease events with triglyceride glucose as continuous variables in Cox models.

| **Incident event** | **Model** | **TyG** | |
| --- | --- | --- | --- |
|  |  | **Hazard Ratio (95%CI)** | ***P*** |
| CVD | Model 1 | 1.79 (1.58-2.04) | < 0.001 |
|  | Model 2 | 1.45 (1.26-1.66) | < 0.001 |
|  | Model 3 | 1.43 (1.24-1.63) | < 0.001 |
| CHD | Model 1 | 2.12 (1.84-2.45) | < 0.001 |
|  | Model 2 | 1.69 (1.44-1.97) | < 0.001 |
|  | Model 3 | 1.63 (1.39-1.90) | < 0.001 |
| Cerebrovascular disease | Model 1 | 1.32 (1.05-1.68) | 0.020 |
|  | Model 2 | 1.11 (0.87-1.43) | 0.402 |
|  | Model 3 | 1.17 (0.91-1.49) | 0.223 |

Model 1 was adjusted by age and sex; model 2 was model 1 plus living alone, current smoking, alcohol consumption, exercise, body mass index, resting heart rate, systolic blood pressure, high-density lipoprotein cholesterol, low-density lipoprotein cholesterol, and diabetic status; model 3 was model 2 with time-varying repeated measures of TyG. TyG, triglyceride glucose; CVD, cardiovascular disease; CHD, coronary heart disease.

**Supplementary Table S3.** Risk of incident cardiovascular disease by quartiles of the TyG index in subgroup analysis

| **Subgroup** | **TyG** |  |  |  | ***P* for trend** | |
| --- | --- | --- | --- | --- | --- | --- |
|  | **Quartile 1 (<8.32)** | **Quartile 2 (8.32-8.61)** | **Quartile 3 (8.61-8.89)** | **Quartile 4 (≥8.90)** |  |  |
| **Men** |  |  |  |  |  |  |
| n | 703 | 787 | 809 | 927 |  |  |
| CVD | 65 (9.25) | 72 (9.15) | 108 (13.35) | 175 (18.88) |  | <0.001 |
| Non-CVD death | 24 (3.41) | 30 (3.81) | 32 (3.96) | 26 (2.80) |  |  |
| Model 1 | 1 (ref) | 0.99 (0.71-1.39) | 1.49 (1.10-2.03) | 2.15 (1.61-2.86) |  | <0.001 |
| Model 2 | 1 (ref) | 0.94 (0.67-1.32) | 1.36 (0.99-1.87) | 1.66 (1.23-2.25) |  | <0.001 |
| **Women** |  |  |  |  |  |  |
| n | 820 | 734 | 706 | 592 |  |  |
| CVD | 56 (6.83) | 56 (7.63) | 69 (9.77) | 104 (17.57) |  | <0.001 |
| Non-CVD death | 17 (2.07) | 17 (2.32) | 22 (3.12) | 15 (2.53) |  |  |
| Model 1 | 1 (ref) | 1.13 (0.78-1.64) | 1.47 (1.03-2.08) | 2.76 (2.00-3.82) |  | <0.001 |
| Model 2 | 1 (ref) | 1.05 (0.72-1.54) | 1.28 (0.90-1.82) | 1.75 (1.24-2.48) |  | <0.001 |
| **Participants without T2DM** |  |  |  |  |  |  |
| n | 1391 | 1382 | 1334 | 1254 |  |  |
| CVD | 105 (7.55) | 114 (8.25) | 151 (11.32) | 208 (16.59) |  | <0.001 |
| Non-CVD death | 39 (2.8) | 40 (2.89) | 46 (3.45) | 29 (2.31) |  |  |
| Model 1 | 1 (ref) | 1.08 (0.83-1.41) | 1.52 (1.18-1.95) | 2.20(1.74-2.79) |  | <0.001 |
| Model 2 | 1 (ref) | 1.00 (0.76-1.31) | 1.35 (1.05-1.73) | 1.62 (1.26-2.08) |  | <0.001 |
| **Participants with T2DM** |  |  |  |  |  |  |
| n | 132 | 139 | 181 | 265 |  |  |
| CVD | 16 (12.12) | 14 (10.07) | 26 (14.36) | 71 (26.79) |  | <0.001 |
| Non-CVD death | 2 (1.52) | 7 (5.04) | 8 (4.42) | 12 (4.53) |  |  |
| Model 1 | 1 (ref) | 0.88 (0.43-1.80) | 1.26 (0.68-2.35) | 2.59 (1.51-4.45) |  | <0.001 |
| Model 2 | 1 (ref) | 0.95 (0.45-1.99) | 1.30 (0.68-2.47) | 2.28 (1.28-4.07) |  | <0.001 |

Values are n (%) or subhazard ratio (95% confidence interval) using the first (lowest) quartile as the referent. Model 1 was adjusted by age and sex; model 2 was model 1 plus living alone, current smoking, alcohol consumption, exercise, body mass index, resting heart rate, systolic blood pressure, high-density lipoprotein cholesterol, low-density lipoprotein cholesterol, and diabetic status. *P* values for linear trend across TyG quartiles were evaluated by a median value within each quartile as a continuous variable. TyG, triglyceride glucose; CVD, cardiovascular disease.

**Supplementary Table S4.** Association of multiple competing-event results across quartiles of triglyceride glucose.

| **Event type** | **N** | **Quartile 1 (<8.32)** | **Quartile 2 (8.32-8.61)** | **Quartile 3 (8.61-8.89)** | **Quartile 4 (≥8.90)** | ***P* for trend** |
| --- | --- | --- | --- | --- | --- | --- |
| non-fatal CHD | 474 | Reference | 1.19 (0.86-1.65) | 1.47 (1.08-2.01) | 2.34 (1.75-3.14) | <0.001 |
| fatal CHD | 26 | Reference | 0.57 (0.13-2.42) | 1.77 (0.58-5.45) | 1.54 (0.46-5.18) | 0.293 |
| non-fatal stroke | 186 | Reference | 0.96 (0.61-1.52) | 1.16 (0.76-1.78) | 1.25 (0.83-1.88) | 0.204 |
| fatal stroke | 48 | Reference | 0.40 (0.16-1.00) | 0.88 (0.41-1.88) | 0.55 (0.22-1.33) | 0.349 |
| non-CVD death | 183 | - | - | - | - |  |

Values are subhazard ratios (95% confidence interval) using the first (lowest) quartile as the referent. The competing model was adjusted by age and sex, living alone, current smoking, alcohol consumption, exercise, body mass index, resting heart rate, systolic blood pressure, high-density lipoprotein cholesterol, low-density lipoprotein cholesterol, and diabetic status. *P* values for linear trend across TyG quartiles were evaluated by a median value within each quartile as a continuous variable. CVD, cardiovascular disease; CHD, coronary heart disease.

**Supplementary Table S5.** Baseline characteristics of the participants according to whether lost to follow up or not.

| **Chharacteristics** | **Final cohort (n=6078)** | **Missing follow-up (n=412)** | ***P*** |
| --- | --- | --- | --- |
| Age (years), mean (SD) | 70.45 ± 6.79 | 63.05 ± 4.18 | <0.001^b^ |
| Women, n (%) | 2852 (46.92) | 196 (47.57) | 0.798^a^ |
| Living alone, n (%) | 1643 (27.03) | 25 (6.07) | <0.001^a^ |
| Current smoking, n (%) | 845 (13.90) | 53 (12.86) | 0.555^a^ |
| Alcohol consumption, n (%) | 754 (12.41) | 36 (8.74) | 0.028^a^ |
| Exercise, n (%) | 602 (9.90) | 41 (9.95) | 0.975^a^ |
| BMI (kg/m2), mean (SD) | 23.74 ± 3.12 | 23.96 ± 2.40 | 0.088^b^ |
| RHR (beats per minute), mean (SD) | 74.54 ± 7.61 | 73.54 ± 5.96 | 0.001^b^ |
| SBP (mmHg) , mean (SD) | 134.09 ± 18.52 | 122.70 ± 11.10 | <0.001^b^ |
| TG (mmol/L) , median (IQR) | 1.38 (1.07-1.70) | 1.45 (1.25-1.62) | 0.145^c^ |
| FPG (mmol/L) , median (IQR) | 5.10 (4.64-5.60) | 5.06 (4.28-5.87) | 0.171^c^ |
| HDL-C (mmol/L) , median (IQR) | 1.33 (1.20-1.69) | 1.49 (1.20-1.89) | <0.001^c^ |
| LDL-C (mmol/L) , median (IQR) | 2.62 (2.14-3.20) | 2.74 (2.11-3.21) | 0.582^c^ |
| TyG index, n (%) |  |  | 0.036^a^ |
| Quartile 1 | 1523 (25.06) | 86 (20.87) |  |
| Quartile 2 | 1521 (25.02) | 121 (29.37) |  |
| Quartile 3 | 1515 (24.93) | 115 (27.91) |  |
| Quartile 4 | 1519 (24.99) | 90 (21.84) |  |
| T2DM, n (%) | 717 (11.80) | 7 (1.70) | <0.001^a^ |

TyG: triglyceride glucose; BMI: body mass index; RHR: resting heart rate; SBP: systolic blood pressure; TG: triglyceride; FPG: fasting plasma glucose; HDL-C: high-density lipoprotein cholesterol; LDL-C: low-density lipoprotein cholesterol; T2DM: type 2 diabetes mellitus. Baseline characteristics were compared between participants lost to follow-up and participants included in the study. ^a^ Indicates Pearson’s Chi-squared test; ^b^ Indicates Welch two sample t-test; ^c^ Indicates Mann-Whitney U test.


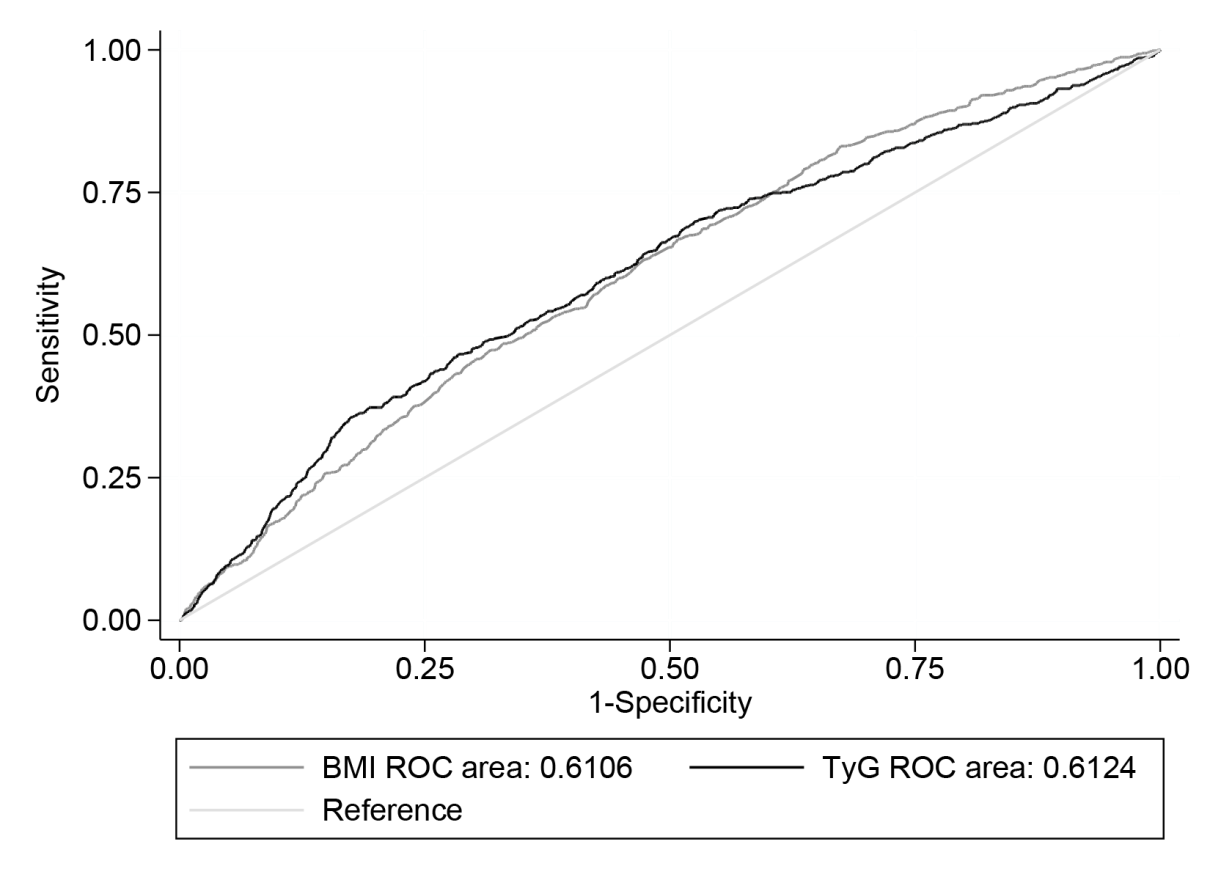


**Supplementary Figure S1**. Receiver operating characteristics (ROC) curves for BMI and baseline TyG predicting cardiovascular disease risk among the elderly people. TyG, triglyceride glucose; BMI, body mass index.

**Supplementary Table S6.** Testing the proportional hazards assumption of a fully adjusted Cox regression model fit.

|  | rho | chi2 | *P* value |
| --- | --- | --- | --- |
| Age (years) | 0.016 | 0.20 | 0.659 |
| Sex |  |  |  |
| Women |  |  | (Reference) |
| Men | 0.050 | 1.72 | 0.190 |
| Living alone |  |  |  |
| No |  |  | (Reference) |
| Yes | -0.025 | 0.48 | 0.490 |
| Current smoking |  |  |  |
| No |  |  | (Reference) |
| Yes | 0.002 | < 0.01 | 0.956 |
| Alcohol consumption |  |  |  |
| No |  |  | (Reference) |
| Yes | 0.053 | 1.96 | 0.161 |
| Exercise (%) |  |  |  |
| No |  |  | (Reference) |
| Yes | -0.005 | 0.02 | 0.895 |
| BMI (kg/m^2^) | -0.018 | 0.19 | 0.661 |
| RHR (beats per minute) | 0.002 | < 0.01 | 0.971 |
| SBP (mmHg) | 0.061 | 2.83 | 0.092 |
| HDL-C (mmol/L) | 0.012 | 0.08 | 0.778 |
| LDL-C (mmol/L) | 0.041 | 1.18 | 0.277 |
| TyG index |  |  |  |
| Quartile 1 |  |  | (Reference) |
| Quartile 2 | -0.006 | 0.03 | 0.866 |
| Quartile 3 | -0.030 | 0.63 | 0.426 |
| Quartile 4 | -0.035 | 0.89 | 0.346 |
| T2DM |  |  |  |
| No |  |  | (Reference) |
| Yes | 0.006 | 0.03 | 0.870 |
| Global test |  | 10.18 | 0.809 |

rho = Spearman's rank correlation coefficient.

**R code**

In this appendix, we provide detailed and annotated R code of multimediator analyses using Aalen additive hazard models and Cox proportional hazard models. Our aim is to assess whether the baseline and follow-up TyG index values mediated the BMI/RHR-CVD relationship in the presence of the mediator-outcome confounders age, sex, smoking, exercise, drinking, SBP, living alone, HDL, LDL, diabetes.

The CVDdata contains the following variables.

| Variables | Explanation |
| --- | --- |
| ID | Subject identifier |
| age | Age at baseline examination |
| sex | Sex (1=man,0=woman) |
| smoking | Smoking status at baseline examination (0=no, 1=yes) |
| exercise | Exercise status at baseline examination (0=no, 1=yes) |
| drinking | Daily alcohol consumption at baseline examination(0=no, 1=yes) |
| SBP | Systolic blood pressure at baseline examination |
| single | Living alone at baseline examination (0=no, 1=yes) |
| HDL | High-density lipoprotein cholesterol at baseline examination |
| LDL | Low-density lipoprotein cholesterol at baseline examination |
| diabetes | Prevalent diabetes mellitus at baseline examination (0=no, 1=yes) |
| RHR | Resting heart rate at baseline examination |
| BMI | Body mass index at baseline examination |
| TyG | Triglyceride glucose index at baseline examination |
| TyGfollowup | Triglyceride glucose index at the final examination |
| t | CVD event time or censor time (years after baseline examination) |
| CVD | CVD event indicator (0=no CVD event, 1=CVD event) |

The R codes are as follows.

# Import data

dat1<-read.csv ("D:/CVDdata.csv", h=T)

library (timereg)

library (survival)

###############

#R code resampling based method to estimate confidence interval and calculate p-value #(mediation_aalen_cox_ci_pval.R). This R file was provided by Huang, Y. T. and Yang, H. I.

#This method was introduced by Huang, Y. T. & Yang, H. I. in 2017 (Causal Mediation Analysis of Survival #Outcome with Multiple Mediators. Epidemiology. 28, 370-378).

###############

source ("mediation_aalen_cox_ci_pval.R")

# A new variable RHRper10 is created.

RHRper10<-dat1$RHR/10

##The 2-mediator Cox model for the association between RHR and CVD.

# For the continuous mediators, we choose linear regressions.

ols_m1<-glm (TyG~age+sex+smoking+exercise+BMI+drinking+SBP+single+HDL+LDL+diabetes+ RHRper10, data=dat1)

ols_m2<-glm (TyGfollowup~age+sex+smoking+exercise+BMI+drinking+SBP+single+HDL

+LDL+diabetes+ RHRper10+TyG, data=dat1)

#The following R code shows the example for a Cox proportional hazards model. If we need the Aalen #additive hazard model, we could use aalen () function and run method = “Aalen”.

cox_m3<-coxph(Surv(t, CVD)~age+sex+smoking+exercise+BMI+drinking+SBP+single+HDL

+LDL+diabetes+ RHRper10+TyG + TyGfollowup, data=dat1)

method ="Cox"

if (method = ="Aalen"){

lambdas < - aalen_m3$gamma

Sigma.lambda<-aalen_m3$robvar.gamma [12:14, 12:14]

} else if (method=="Cox"){

lambdas<-cox_m3$coef

Sigma.lambda<-cox_m3$var [12:14, 12:14]

}

alphas<-ols_m2$coef

Sigma.alpha<-summary (ols_m2)$cov.scaled [13:14, 13:14]

deltas<-ols_m1$coef

Sigma.delta<-summary (ols_m1)$cov.scaled [13, 13]

mediation_ci2(lambdas[12], lambdas[13], lambdas[14], Sigma.lambda,

alphas[13], alphas[14], Sigma.alpha, deltas[13], Sigma.delta, G=10^6,

method=method)

#Finally, the output includes the path-specific Effects and total effects of RHR (10 unit increase) on the CVD #risk mediated through the baseline TyG and the follow-up TyG. Here the output is based on the results from #the Cox proportional hazard model. Thus, the estimates are the hazard ratio, confidence interval and P-value. #SY indicates RHR-CVD pathway; SGY indicates RHR-TyG2 (follow-up triglyceride glucose)-CVD #pathway; SMY indicates RHR-TyG1(baseline triglyceride glucose)-TyG2-CVD pathway; TE indicates the #total effect.

#[1] "SY:"

#[1] 1.238302

# 2.5% 97.5%

#1.129521 1.357460

#[1] "pval_SY= 6e-06"

#[1] "SGY:"

#[1] 1.01166

# 2.5% 97.5%

#1.003455 1.021134

#[1] "pval_SGY= 0.017632"

#[1] "SMY:"

#[1] 1.014454

# 2.5% 97.5%

#1.007039 1.023650

#[1] "pval_SMY= 0.003512"

#[1] "TE:"

#[1] 1.270848

# 2.5% 97.5%

#1.158730 1.393747

#[1] "pval_TE= 0"
